# Resection Margins in Head and Neck Cancer Surgery: An Update of Residual Disease and Field Cancerization

**DOI:** 10.3390/cancers13112635

**Published:** 2021-05-27

**Authors:** Annouk S. Pierik, C. René Leemans, Ruud H. Brakenhoff

**Affiliations:** Amsterdam UMC, Vrije Universiteit Amsterdam Tumor Biology and Immunology Section, Otolaryngology-Head and Neck Surgery, Cancer Center Amsterdam, 1081 HV Amsterdam, The Netherlands; a.pierik@amsterdamumc.nl (A.S.P.); cr.leemans@amsterdamumc.nl (C.R.L.)

**Keywords:** head and neck squamous cell carcinoma, residual disease, field cancerization, recurrence, second primary tumor, molecular diagnosis, leukoplakia

## Abstract

**Simple Summary:**

Curative treatment of head and neck squamous cell carcinoma (HNSCC) is largely dependent on locoregional control of the disease. However, HNSCCs frequently recur, and even after surgery with histologically tumor-free surgical margins, tumors may relapse in approximately 10–30% of patients. The development of local relapse despite the fact that surgical margins were tumor-free relates to two independent pathobiological mechanisms: minimal residual disease and field cancerization. Here, we outline the cellular and biological background of local relapse that resulted in a most recently reported clinical trial. We further discuss directions that may improve treatment results in the future.

**Abstract:**

Surgery is one of the mainstays of head and neck cancer treatment, and aims at radical resection of the tumor with 1 cm tumor-free margins to obtain locoregional control. Surgical margins are evaluated by histopathological examination of the resection specimen. It has been long an enigma that approximately 10–30% of surgically treated head and neck cancer patients develop locoregional recurrences even though the resection margins were microscopically tumor-free. However, the origins of these recurrences have been elucidated by a variety of molecular studies. Recurrences arise either from minimal residual disease, cancer cells in the surgical margins that escape detection by the pathologist when examining the specimen, or from precancerous mucosal changes that may remain unnoticed. Head and neck tumors develop in mucosal precursor changes that are sometimes visible but mostly not, fueling research into imaging modalities such as autofluorescence, to improve visualization. Mostly unnoticed, these precancerous changes may stay behind when the tumor is resected, and subsequent malignant progression will cause a local relapse. This led to a clinical trial of autofluorescence-guided surgery, of which the results were reported in 2020. This review focuses on the most recent literature of the improved diagnosis of the resection margins of surgically treated head and neck cancer patients, the pathobiological origin of recurrent disease, and relevant biomarkers to predict local relapse. Directions for further research will be discussed, including potential options for improved and personalized treatment, based on the most recently published data.

## 1. Introduction

Head and neck squamous cell carcinoma (HNSCC) develops in the epithelial lining of the oral cavity, pharynx, larynx and cervical esophagus. HNSCC comprises approximately 5% of all newly diagnosed cancer cases in developed countries, and the incidence is rising. Head and neck cancer is one of the more common cancers in the world, leading to approximately 900,000 new cases and 500,000 deaths annually [1,2].

HNSCC is caused by classical risk factors such as tobacco use, excessive alcohol consumption, persistent infection with human papillomavirus (HPV) and genetic predisposition [3,4]. In particular, oropharyngeal squamous cell carcinomas (OPSCC) are caused by high-risk HPV infection, and HPV-positive OPSCC have a very favorable prognosis.

Treatment of HNSCC depends on the site and stage of disease. Early-stage tumors can generally be treated with single modality therapy encompassing either surgery or radiotherapy. However, the majority of HNSCC patients present with more advanced stages of disease. For these patients, a multimodality therapy is applied and may consist of definitive chemoradiotherapy, the concomitant application of systemic cisplatin-based chemotherapy with locoregional radiotherapy. Additionally, for patients unfit to receive cisplatin, the combination of cetuximab with radiotherapy could be practiced [5]. For tumors in the oral cavity, upfront surgery is generally applied, combined with postoperative (chemo)radiotherapy, depending on the stage of disease, imaging parameters, and histological findings after surgery [6]. Treatment of HPV-positive oropharyngeal tumors generally consists of primary (chemo)radiotherapy without upfront surgical intervention. However, in recent years, transoral robotic surgery is being applied for, e.g., base of tongue tumors, which has refueled interest in surgical margin research of HPV-positive disease.

The diagnosis, treatment and surveillance of HNSCC have improved over the years, and it is therefore somewhat striking that the 5-years survival of more advanced stage tumors gradually increased but seems to have reached a plateau at approximately 60% [7]. To put this in broader perspective, the improvement in survival may even in part relate to the increasing incidence of HPV-induced OPSCC and associated favorable prognosis [4]. Hence, the advances in clinical management apparently do not directly translate into survival benefits, although improved function and quality of life are reported and are important as well [7,8].

Despite the intense treatment protocols applied, locoregional recurrences still occur in 30–40% of advanced stage patients and are difficult to manage, as these are mostly detected late. In addition, patients are at risk of developing second primary tumors, both in the head and neck region as well as in the lungs. The clinical distinction between a local recurrence and a second primary tumor in the same or adjacent anatomical region is based on the distance and the time interval. A relapse is indicated as a local recurrence when it arises within 2 cm of the location of the index tumor and within 3 years [9]. Relapses not fulfilling these criteria are termed second primary tumors, and these may occur simultaneously but also even after a decade.

**Figure 1:**
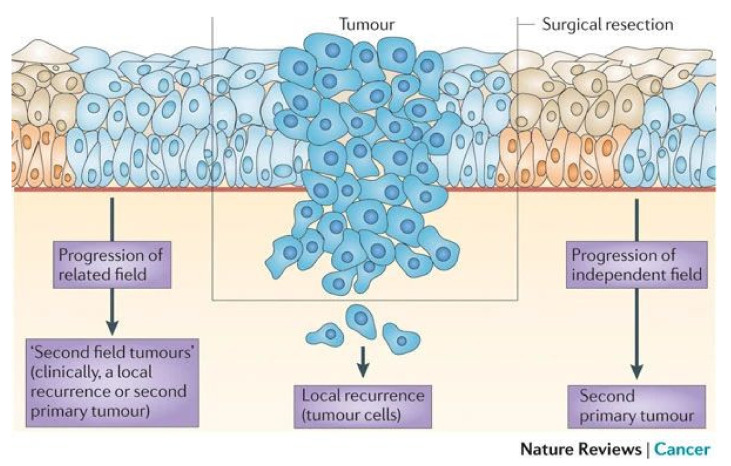
Taken from Leemans, C.R. et al. [11]. Schematic representation of the pathobiological origin of local relapse. A tumor has developed in the light blue preneoplastic field, is diagnosed and resected. Minimal residual disease is shown in the middle and may escape histological examination, leading to local recurrence. Additionally, the preneoplastic field can stay behind unnoticed when extending into the surgical margins. Progression of the remaining preneoplastic field could lead to a local relapse clinically diagnosed as local recurrence or second primary tumors, depending on the distance and time interval. At the right-hand side, the progression of an independent field is shown, which could also progress into a second primary tumor.

The development of local and regional recurrences are a persistent problem, and most are unexpected as patients are treated by radical surgery with the ultimate aim of completely removing the tumor, and when appropriate the involved lymph nodes in the neck. The surgical specimen is transferred to a pathology laboratory and screened by microscopy in a standardized fashion to confirm that the tumor has been removed completely. In addition, the level of lymph node metastasis and extranodular spread is reported. Remarkably, even when the surgical margins are histologically tumor-free, local recurrences may still occur in 10–30% of patients. Molecular research focused on the origin of these unexpected local relapses, and revealed that these can be explained by two pathobiological mechanisms that are displayed in Figure 1. The first mechanism relates to a phenomenon referred to as minimal residual disease, also known as minimal residual cancer. Despite the fact that surgery aims at the radical resection of the tumor with 1 cm margins to obtain local control, and removing all tumor-involved lymph nodes to obtain regional control, tumor cells may stay behind unnoticed, even after microscopic examination of the resected specimen [10]. It should be noted that these 1 cm tumor-free margins are mainly applicable to oral cavity cancers; margins of 1 cm are not always possible at other sites such as the oropharynx and larynx, and a smaller margin is often accepted [6]. Tumor cells that remain in the margins after surgery appear to be too small in number to be detected by routine histopathology, and may eventually develop into a recurrence. To treat these cells, postoperative radiotherapy or even postoperative chemoradiotherapy is applied, but this has to be decided based on gross tumor characteristics and the likelihood of developing a recurrence.

In addition, there is a second pathobiological mechanism in play, which relates to the carcinogenesis of these tumors. HNSCCs develop in precancerous mucosal changes characterized by tumor-associated genetic changes and often morphological changes indicated as dysplasia. These premalignant mucosal changes, also coined as ‘fields’ in line with the field cancerization concept, are mostly not visible to the naked eye, may have dimensions up to centimeters in diameter, and consequently often surround the primary tumors. Not being visible to the naked eye, they can stay behind unnoticed after surgical excision of the tumor, and may eventually progress into a local relapse. The premalignant changes can be visualized by autofluorescence (see below), and in 2020, the results of autofluorescence-guided surgery were reported, indicating how to proceed in the future with novel interventions.

## 2. Minimal Residual Disease

Minimal residual disease is defined as tumor cells that remain present after surgery but are not detected by routine diagnostic methods, and may consist of isolated cells or small tumor deposits. The detection of minimal residual disease is important for treatment planning and estimating prognosis. The major factor that explains why these cancer cells remain undetected is the problem of undersampling: when few tumor cells are present in a large tissue volume, detection of these cells in a single or even a few pathological sections becomes very difficult. This is the reason that sentinel lymph node biopsies are investigated by stepwise sectioning to increase the chance of tumor detection. The sentinel node biopsy concept assumes that the first draining lymph node, diagnosed by radioactive tracer injection around the tumor, is indicative of the status of the neck, and it is therefore biopsied. Obviously sensitive and specific detection of tumor cells in the excised node is then key. Particularly isolated cells are easily overlooked, and besides stepwise sectioning, immunostaining may be of help, which is indeed also applied in the histological analysis of sentinel node biopsy specimen.

Imaging methods such as MRI, CT and FDG-PET lack the sensitivity for minimal residual disease detection. FDG-PET is based on the increased glucose metabolism and over-expression of glucose transporters in tumors which are targeted by F-18-fluorodeoxyglucose (FDG), which is the most frequently used PET tracer. In several studies, it has been shown that a negative result of FDG-PET is very reliable, with a negative predictive value for the presence of malignancy of 95% [12,13]. However, in these studies, histologically detectable tumor was used as gold standard, tumor loads that do not compare to minimal residual cancer. Accuracies for minimal residual disease detection cannot be determined and will be much lower. For a PET signal, a minimal tracer uptake is required and generally refers to millions of tumor cells already. In addition, tissue after treatment is often inflamed, resulting in increased uptake of FDG, causing a false signal and resulting in a decreased specificity of FDG-PET. Other routine imaging modalities such as MRI and CT are also too insensitive for minimal residual disease detection, and require tumor deposits of 0.5–1 cm in diameter. Minimal residual disease consisting of a few tumor cells cannot be detected by any external imaging method.

It is clear that there is a need for improved methods to detect residual disease, and it is of importance as it could be employed to determine post-operative management. In the following section, the different methods for the detection of minimal residual disease will be highlighted. Topics include molecular diagnostic approaches and intraoperative imaging.

### 2.1. Detection Methods of Minimal Residual Disease

One of the very first molecular diagnostic approaches to detect minimal residual disease was reported by Brennan et al., in which they studied *TP53* mutations in the margins as molecular marker [14]. Superficial margins containing mucosal cells and deep margins were analyzed for mutant *TP53* as a biomarker for residual disease. Mutant *TP53* was analyzed by differential hybridization PCR fragments cloned in bacteriophage vectors, the so-called plaque assay, an extremely laborious approach. Next generation sequencing has become a game changer in this respect. Detection of mutant *TP53* in the surgical margins was associated with a high risk of locoregional recurrence, at the time assumed as being caused by residual tumor cells. The initial results from Brennan et al. were underpinned by more recent studies [15,16]. However, in subsequent studies, it became apparent that the detection of mutant *TP53* in surgical margins does not always indicate minimal residual cancer cells at all. The mutations in *TP53* are an early genetic change in head and neck carcinogenesis and could also be part of a precancerous field, specifically in the superficial margins. It is important to be able to differentiate between residual disease and a precancerous field. Residual cancer cells should be treated, for example by post-operative radiotherapy, which is not indicated for a precancerous field. The concept of precancerous fields and field cancerization is further described in Section 3 of this review.

Besides mutations, epigenetic changes, most particularly DNA methylations that frequently occur in cancer, including head and neck cancer, have also been tested [17]. During carcinogenesis, the epigenetic landscapes of the premalignant cells change. These changes could include differential DNA methylation, modification of histones, nucleosome positioning and higher orders of chromatin changes. These changes give growth advantages and will contribute to cancer-associated phenotypes [18]. Shen et al. reasoned that the detection of minimal residual disease after treatment requires a greater analytical sensitivity and is beyond the technical limits of mutation-based ctDNA analysis, and that specific enrichment of methylated DNA fragments from cfDNA could be a solution [19]. In theory, methylation markers are generally detected by PCR-based assays, and are very sensitive. However, specificity is a major problem with assays using methylation markers as epigenetic changes are dynamic and not so definitive as somatic mutations.

In addition to DNA-based methods using somatic mutations or methylation as markers, RNA-based techniques have been evaluated to improve the detection of minimal residual disease, now specifically focused on analysis of the deep surgical margins. One of the genes with high expectations was hLy6D, a gene highly and specifically expressed in squamous cells. As only deep margins were tested, expression in normal or dysplastic mucosa could be ignored. Graveland et al. found initially that the application of hLy6D qRT-PCR to detect minimal residual cancer in surgical margins appeared to have potential, but in a subsequent prospective study, the authors failed to demonstrate any prognostic relevance of hLy6D detection [16]. A major problem again appeared to be undersampling. Even when analyzing multiple samples from the deep margins, up to five or more, many cases developing a recurrence were missed. Hence, the problem of undersampling that hampers histopathology seems also to hamper molecular approaches.

Is there a way to circumvent undersampling of margins for detecting residual disease? An alternative approach may be the use of liquid biopsy, and several markers have been evaluated to determine the presence of residual disease in samples of blood and saliva. A potential biomarker is the presence of circulating tumor DNA (ctDNA). CtDNA is cell-free DNA that is shed from tumor cells into the circulatory system or other bodily fluids, and consequently carries tumor-associated somatic mutations. The preferred techniques for ctDNA detection are next generation sequencing (NGS) methods using either target-enrichment DNA sequencing or PCR amplicon assays that allow high read coverage, which is essential because the mutant allele fractions are low [20,21].

The added value of ctDNA analysis by using target enrichment sequencing, a commercial workflow termed CAPP-seq, for early detection of recurrent disease was demonstrated by Chaudhuri et al. in lung cancer patients [22]. In this study, positive ctDNA samples 4 months after treatment identified the patients who would relapse with a sensitivity of 94% and a specificity of 100%. This was followed by the study of Hellmann et al., using the same CAPP-seq technique to perform ctDNA analysis, to assess the risk of progression after long-term response to PD-(L)1 blockage in non-small cell lung cancer (NSCLC) [23]. These authors demonstrated that analysis of ctDNA as a surveillance tool in patients with advanced NSCLC undergoing PD-(L)1 blockage therapy can be highly accurate for detecting minimal residual disease and predicting eventual progression. All patients with detectable ctDNA eventually progressed during follow-up (Fisher *p* < 0.0001; positive predictive value = 1, 95% confidence interval (CI), 0.51–1; negative predictive value = 0.93 (95% CI, 0.80–0.99)). Furthermore, the clinical significance of the detection of residual disease by ctDNA analysis was also shown in other studies from other malignancies such as breast, colon and bladder cancer [22,24,25,26,27,28].

Only a few authors studied the added value of ctDNA analysis specifically in HNSCC. Most recently, Mes et al. developed a method for the combined analysis of copy number alterations (CNAs), HPV-DNA and somatic mutations in ctDNA from plasma [21]. Samples before treatment of 40 HNSCC patients were used for the analysis; this resulted in a detection HPV-DNA in 100% of the plasma in HPV-positive cases, and CNAs in 52% and somatic mutations in 67% of the plasma samples in all cases. Other studies showed comparable results within plasma samples of HNSCC patients. Schwaederle et al. showed a detection rate of 88%, Schirmer et al. showed a detection rate of 74%, and Galot et al. showed a detection rate of 51% [29,30,31]. Wang et al. performed target-enrichment sequencing of recurrently mutated genes and HPV in tumor-derived DNA from saliva and plasma samples from 93 patients [32]. The ctDNA in saliva could be detected in 47–100% of the cases and in plasma in 80–100% of the cases. Furthermore, it was interesting that in a subgroup of nine cases, ctDNA was detected post-treatment and before clinical relapse in all patients who developed a recurrence. The other cases without ctDNA in the studied samples remained disease-free. Although these results are promising, ctDNA analysis seems not ready to be implemented in clinical care, and it is at present unclear whether it can be applied either as a method for minimal residual disease detection to allow clinical decision making for post-operative management, or as a monitoring tool during follow-up for early detection of recurrent disease. CtDNA is present in low concentrations in the plasma and blood; hence, the detection methods must be sensitive enough for the detection of mutations at mutant allele fractions of <0.1% on the basis of only a few mutant DNA strands, so methods have to be optimized to perfection. In addition, the detection of ctDNA should be interpreted with caution, somatic mosaicism such as clonal hematopoiesis of indeterminate potential (CHIP) might cause false positive results, particularly when the mutations in the tumor are not determined [33]. Well-designed longitudinal studies with structured sampling will reveal whether ctDNA detection could give direction to personalized post-operative therapy planning or should be reserved for disease monitoring.

Other potential biomarkers for liquid biopsy analysis are epigenetic-based. Shen et al. developed the cell-free methylated DNA immunoprecipitation and high output sequencing (cfMeDIP-seq) approach for genome-wide bisulfite-free plasma DNA methylation profiling. The cfMeDIP-seq was compared to ultradeep hybrid-based capture sequencing, and the limits of detection were 0.001% and 0.1%, respectively. In a large prospective case–control sub-study, which is part of the circulating Cell-free Genome Atlas, the performance of targeted methylation analysis of circulating cell-free DNA was assessed to detect and localize multiple cancer types across all stages with a high specificity of 99.3% [18]. Detection increased with increasing stage: for a group of patients in stage I, the sensitivity dropped to 39%. Further evaluation of this test is needed in prospective population-level studies to evaluate clinical utility.

Analysis of saliva is, compared to the blood-based techniques, an elegant method to non-invasively detect biomarkers in HNSCC patients. Romani et al. analyzed the global miRNA expression in OSCC patients by using quantitative real-time polymerase chain reaction (qRT-PCR) assays and compared these to the results of healthy controls [34]. Three miRNAs (miR-106b-5p, miR-423-5p and miR-193b-3p) were expressed in the saliva at high levels in OSCC patients. Furthermore, these authors demonstrated that miR-423-5p could assist risk stratification in OSCC patients. An overview of a number of detection methods for minimal residual disease in surgical margins or liquid biopsies is shown in Table 1.

### 2.2. Treatment of Minimal Residual Disease

The presence of macroscopic or microscopic residual disease in the margins appears to be a poor prognostic feature [10]. According to the EHNS-ESMO-ESTRO clinical practice guidelines, postoperative radiotherapy is recommended for patients with macroscopic (R2) or microscopic (R1) disease in the surgical margins [6]. Patients with macroscopic residual disease should best undergo re-resection or receive definitive (chemo)radiotherapy. It has also been shown that re-resection of initially tumor-positive margins (mucosal or deep) will result in comparable locoregional control and overall survival rates with patients with initial clear resection margins [6]. Ferris at al. conducted a phase II trial of post-operative radiotherapy with concurrent cisplatin combined with panitumumab, targeting the EGFR pathway, in high-risk patients after resection of their head and neck cancer [35]. In the trial, patients at high risk for locoregional recurrence were enrolled with resected tumors with pathologic stage III or IVa HNSCC (HPV-negative), without gross residual tumor, margins <1 mm, extracapsular extension, perineural or angiolymphatic invasion or more than 2 positive lymph nodes. The treatment consisted of radiotherapy (60–66 Gy over 6–7 weeks) combined with 30 mg/m^2^ of cisplatin and 2.5 mg/kg of panitumumab weekly. A progression-free survival rate of 70% was reported after two years. Another optional treatment is the use of checkpoint inhibitors such as Nivolumab, an anti-PD-1 monoclonal antibody. Nivolumab was studied in different trials using patients with recurrent or metastatic HNSCC [36,37]. Patients receiving Nivolumab had a longer progression free survival and experienced fewer toxic effects compared to standard of care. Hence, when reliable molecular detection methods are developed for the detection of minimal residual disease, clinical decision making on post-operative treatment comes into play. Patients with positive molecular margins should be treated by post-operative (chemo)radiotherapy or alternative regimens with immunotherapy as indicated, while patients with negative molecular margins could be spared postoperative adjuvant treatment.

## 3. Field Cancerization

After minimal residual disease, the second proposed mechanism that explains the development of local recurrences even when the surgical margins are tumor-free is ‘field cancerization’. HNSCC is preceded by precancerous changes in the mucosal epithelium characterized by tumor-associated genetic changes, also termed precancerous fields. Typical genetic changes that are identified are losses of chromosome arm 9p21 encompassing the *CDKN2A* gene encoding the p16^Ink4A^ cell cycle inhibiting protein, and/or loss of 17p13 encompassing the *TP53* gene. Additionally, mutations in *NOTCH1* and *FAT1* have been reported [38]. These are also genetic changes frequently found in HNSCC.

Some of the precancerous changes are macroscopically visible [39]. Leukoplakia is the most common visible precancerous lesion that may precede invasive carcinomas. Standard clinical policy is to take a biopsy to exclude invasive growth, which also allows grading for epithelial dysplasia on the basis of morphological abnormalities. Dysplasia is the best predictor of malignant transformation of leukoplakia, particularly when ‘differentiated dysplasia’, a novel morphological abnormality, is added [40].

However, most fields are not macroscopically visible, but are detected in resection specimens by means of microscopic examination of surgical margins as epithelial dysplasia, and are graded as mild, moderate and severe. Detection and grading of dysplasia in the surgical margins is not very accurate to predict recurrent disease in treated cancer patients, but the very new morphological classification of ‘differentiated dysplasia’ that also appeared to be very informative for risk assessment of leukoplakia, might be a game changer for risk assessment of surgical margins as well.

The precancerous fields can be much larger than the primary tumor and generally have a normal macroscopic appearance. Because of the dimensions of these fields and the fact that most are not visible to the naked eye, they often stay behind when the tumors are excised. A second malignant transformation in such a field that stayed behind unnoticed within 2 cm of the location of the index tumor and within 3 years will be diagnosed as a local recurrence. Alongside the development of local recurrences, the field cancerization concept also explains the development of second primary tumors: when they arise more than 2 cm away of the index tumor or after 3 years. Other second primary tumors may arise independently.

The first description of the concept of field cancerization was made by Slaugter et al. in 1953 [4]. In this study, OSCC tumors of 783 patients were reviewed by microscopy, and independent multiple tumors were found in 11.2%. The frequent development of local recurrences and second primary tumors in the oral cavity was explained by the abnormal, hyperplastic and often atypical epithelium surrounding the tumors. In more recent years, when the research field of cancer genetics emerged, carcinogenesis was described in terms of accumulating genetic changes, and also the field cancerization concept was described in relation to genetic changes [17]. Braakhuis et al. proposed a definition based on molecular findings, and they defined field cancerization as genetically altered cell growth of a monoclonal origin, giving rise to a contiguous pre-cancerous field [41].

To further investigate the characteristics of preneoplastic cells, first Van Zeeburg et al. and later De Boer et al. brought biopsies surrounding HNSCCs into culture and established a variety of preneoplastic cultures that were genetically characterized. Remarkably, 50% of the cultures contained genetic changes, either mutations, copy number alterations, or both. Some were indistinguishable from invasive carcinomas, but never displayed growth when injected into immune deficient mice, while tumor cells do this very frequently [38,42].

### 3.1. Detection Methods of Precancerous Fields

For the detection of a preneoplastic field, the mucosal epithelium of the resection margins of the primary tumor can be assessed by a variety of molecular methods. These range from routine dysplasia grading, which is less informative, to immunostaining, mutation analyses, and the detection of copy number changes, e.g., by loss of heterozygosity analysis using microsatellite PCR [43]. Additionally, methylation and expression profiling are options for detection and risk assessment of precancer [44,45]. Likewise, methylation marker analyses might serve as an early indicator of recurrent disease [46].

The initial studies on the genetic changes associated with malignant transformation of the mucosal epithelium and field cancerization came from Califano et al. These authors used loss of heterozygosity (LOH) analysis by microsatellite PCR, the state of the art at that time, to assess genetic changes in tumors and dysplastic epithelium to porose the first genetic progression model of HNSCC [47]. Tabor et al. determined loss in heterozygosity in tumors and three to five noncontiguous mucosal biopsies surrounding these tumors of 28 HNSCC patients using different microsatellite markers at 9p, 3p and 17p, and described in detail the process of field cancerization for the very first time in genetic terms [48]. Genetically altered fields were identified in 36% of the patients, and, importantly, in 25% of patients these extended into the surgical margins. Later, it was shown that approximately half of the local recurrences and half of the second primary tumors in the same or adjacent anatomical area related to field cancerization, demonstrating the clinical relevance of field cancerization.

Many other molecular markers have been investigated to detect these fields and predict relapse, such as eukaryotic translocation initiation factor 4E (eIF4E) and *TP53*, which can be determined by immunohistochemical analysis [49]. Despite successful results, eIF4E was not studied by many others, likely due to the fact that immunostaining of these markers demands extensive optimization in order to allow discrimination between high-risk and low-risk margins.

A major factor impeding progress in the studies on field cancerization is the fact that the majority of fields are not visible to the naked eye, which hampers diagnosis but also therapeutic interventions. There are two separate developments to approach this problem. On the one hand, visualization tools are being developed and tested including autofluorescence, narrow band imaging, Raman spectroscopy and many others. An interesting option is narrow band imaging (NBI). The concept is based on the specific wavelengths that are absorbed by hemoglobin, and consequently NBI visualizes the vascular network of a lesion [50]. Hence, precancerous mucosal changes with increased angiogenesis can be detected with NBI. In a prospective study with 91 patients, images were acquired of oral premalignant and malignant lesions under white and NBI light prior to biopsy for histological diagnosis [51]. NBI was applied on lesions with different histopathologic grades, and were compared to the histological diagnosis. Sensitivity and specificity depended on histopathologic grade or stage of disease, and ranged from 63 to 99% or 89 to 100%, respectively. The accuracy of NBI to detect less severe dysplastic stages in this study suggested that NBI has potential for surveillance. A second interesting alternative is autofluorescence detection. Mucosal cells that show genetic and molecular abnormalities may change their autofluorescence properties, which can be visualized with the proper detection probes. Poh et al. showed the potential of autofluorescence [52], and moved this research program to intervention studies (see below).

The alternative approach for imaging methods is to brush cells from the mucosal surface and test these for alterations in methylation and genetic markers or morphological changes by means of cytology. Adequate detection of precancerous fields was shown using genetic analysis by microsatellite PCR of exfoliated cells removed by a brush [16,53]. This assay on the basis of the loss of heterozygosity had an initial sensitivity and specificity of, respectively, 78% and 100% when compared to the presence of genetic changes in the biopsy specimen, but the sensitivity dropped to 45% in a subsequent larger validation study, while the specificity remained at 100%. In other studies, the application of exfoliative cytology was analyzed using brush biopsy. These methods also compared brushed cells, analyzed by microscopy, with the gold standard of tissue biopsy for the detection of oral premalignant lesions [54]. The performance of cytology was tested on 117 clinically diagnosed precancerous lesions. The slightly modified brush biopsy, a noncomputerized assisted analysis of a brush biopsy sample obtained by a toothbrush, showed the highest sensitivity of 81% and a specificity of 68%. Brush biopsies and exfoliative cytology are interesting options for the detection premalignant lesions during follow-up for monitoring patients, because sampling can take place at the outpatient clinic. However, the lack of visibility of precancerous changes remains a hurdle. Of note, biopsy is at present still needed to exclude invasive growth and confirm the diagnosis. An overview of a number of detection and visualization methods for field cancerization is shown in Table 2. 

### 3.2. Treatment Options

The major challenge for intervention strategies is to prevent a second malignant transformation of the precancerous fields, and the development of a local relapse. Severe dysplasia in surgical margins is a soft indicator for post-operative radiotherapy, but mild and moderate dysplasia in the margins are not considered in management. Resection is not an option as most fields are not visible, while post-operative radiotherapy might not even be effective. Radiotherapy is therefore not indicated. Hence, what kinds of interventions are possible? Due to the given possibility but not certainty of malignant progression, proposed treatments should have minimal toxicity and minimal adverse effects. The possibility of removing the precancerous field by surgical excision, laser surgery or cryotherapy seems tempting, but in that case visualization is key. Additionally, even then, many studies have shown that excision of visible lesions such as leukoplakia does not reduce the risk of malignant formation [39,55]. Leukoplakias recur or tumors develop elsewhere in the same or adjacent anatomical region.

As indicated above, autofluorescence detection has been used to improve the visualization of precancerous changes. Using a simple tool, autofluorescence (AF) can be detected in the clinic, and this even led to a clinical trial of AF-guided surgery, of which the results were reported very recently [52]. In this study, 457 patients with histologically confirmed high-grade dysplasia/carcinoma in situ or T1 to T2 OSCC were randomized between standard white light surgery or AF-guided surgery, the latter including resection of tissue with abnormal AF [56]. Remarkably, the observed 3-year local recurrence rate was higher in the AF group, indicating that excision based on AF seems to have no added value in preventing local relapse. This is a very recent disappointing insight that sets back the development of effective interventions. Obviously autofluorescence may not be the optimal method for the visualization of precancerous mucosal changes, and improved techniques may change this. Nevertheless, there is also a possibility that surgical removal remains ineffective even with improved visualization of the precancerous change. This result fits with the experience of removing leukoplakia lesions, which is also not effective to prevent cancer. The invisible precancerous changes may recur as is observed for leukoplakias as well, and most likely multiple precancerous changes are present in patients. Removing one is then not effective. Most likely, systemic treatment is in that situation a more promising option.

The alternative therefore, is to treat these high-risk precancerous changes using chemical or biological compounds, formerly termed chemoprevention. To improve the clinical outcome of HNSCC and leukoplakia patients by preventing malignant transformation of precancerous fields, many chemopreventive agents have been studied over the years. Chemoprevention is defined as an approach involving applying natural, synthetic or biological agents to prevent, reverse or suppress carcinogenic progression [57]. When field cancerization was described in terms of genetic changes, this can be translated as the treatment of precancerous changes. Retinoids (synthetic vitamin A) were among the first agents to be studied in this context. The rationale was that retinoids could reverse the abnormal differentiation of epithelial cells that result from vitamin A deficiency [58]. In a subsequent phase III randomized controlled trial, 1190 early-stage HNSCC patients were assigned to either 13-cisretinoic acid or a placebo for a period of 3 years, with a follow-up of four years [59]. The authors reported no evidence for improved survival (hazard ratio (HR) = 1.03, 95% confidence interval (CI) = 0.81 to 1.32) or reduction in second primary tumors (HR = 1.06, 95% CI = 0.83 to 1.35). In a subsequent study with retinyl palmitate and N-acetylcysteine, the same negative result was obtained [60]. Hence, retinoids seem to be not the agents of choice.

Besides retinoids, EGFR inhibitors have been explored as chemopreventive therapy. The epidermal growth factor receptor (EGFR) plays an active role in the growth and survival of HNSCC. Premalignant lesions also show a high level of EGFR expression and might benefit from EGFR blockade to prevent malignant progression. A monoclonal anti-EGFR antibody, cetuximab, was studied in a phase II trial by Khan et al. [61]. Seventeen patients with high-risk premalignant lesions in the upper aerodigestive tract were included and randomized to treatment with cetuximab or observation, with the option for crossover to cetuximab therapy for patients in the control group. In the results, a trend towards a decrease in dysplasia grade was seen in the cetuximab treated group although this was non-significant (*p* = 0.082). Next to cetuximab, erlotinib, a small molecule EGFR kinase inhibitor, has been studied, but positive clinical results were not reported as well [62]. Hence, EGFR inhibitors seem to be not the first choice at present.

The establishment of precancerous cultures allowed researchers to follow a more rational approach. Cultures of precancerous cells, indicated as precancer based on their genetic changes, were exploited in high-throughput functional genetic screens using siRNA and CRISPR/Cas9 technologies to search for druggable target genes. The first possible druggable gene that was identified was Polo-like kinase 1 (*PLK1*) [63]. *PLK1* was identified by an array-based screening of a customized library of tumor-lethal small interfering RNAs (siRNAs), identified in previous genome-wide screens in HNSCC tumor cell lines. PLK1 appeared to be essential for the survival of squamous cancer as well as preneoplastic cells, while normal cells do not respond to PLK1 inhibition. PLK1 inhibition caused monopolar spindles and mitotic catastrophe in (pre)cancer cells. The results of other studies supported this observation [64]. A second target, Wee1-like kinase (*WEE1*), was identified following the same approach and was found as a very promising target for both tumor and precancerous cells. WEE1 (as well as PLK1) is inhibited by adavosertib, and inhibition in cancer cells causes cell death in mitosis due to the induction of DNA damage and unsupervised entry of mitosis. Hence, inhibitors of WEE1 and PLK1 have promise as systemic interventions to treat precancerous changes.

In this era of immune therapy with checkpoint inhibitors, these have also been considered to treat precancerous changes in different preclinical and clinical studies. Previous studies revealed that immune checkpoint inhibitors targeting the interaction of programmed death receptor 1 (PD-1) on T-cells with the PD-1 ligand PD-L1 on cancer cells improved the survival of patients with recurrent/metastatic HNSCC [37,65]. Wang and colleagues used the 4-nitroquinoline-1-oxide (4-NQO) mouse model of oral carcinogenesis to test if PD-(L)1 blockade also inhibits the progression of oral premalignant lesions. Mice were randomized to either drinking water with a blocking antibody for PD-1 or IgG as control. Their results show that anti-PD-1 antibody decreased the formation of oral dysplastic lesions, prevented their progression to SCC and induced specific patterns of expression of immune-modulatory receptors on the T cell infiltrates of oral premalignant lesions [66]. In a similar setting with a 4-NQO mouse model, Monteiro de Oliveira Novaes et al. studied the impact of targeting different pathways on the development of oral premalignant lesions [67]. The most effective treatment to reduce the progression to OSCC was accomplished by targeting CD40 with an agonist monoclonal antibody. PD-1/PD-L1 pathway blockade also reduced the progression to OSCC, although less effective. The utility of PD-1 inhibition and targeting CD40 could be useful, although we need to keep in mind that treatments with antibodies are expensive and could cause toxicities.

Future research for systemic treatment of premalignant lesions could focus on immune intervention. In addition, it will remain important to study the molecular pathways that can be targeted to prevent malignant progression. Moreover, precancerous changes may be heterogeneous. Different authors classified oral pre-malignant lesions into different subgroups based on gene expression profiles, and performed risk assessment for malignant progression [68,69]. Foy et al. identified two main oral premalignant lesions gene-expression subtypes, immunological and classical, in 86 lesions studied. A multivariate analysis showed that decreased miRNA-142-5p expression, and lower T-cell, monocytic and myeloid dendritic cells (MDC) immune infiltration were all significantly associated with oral cancer development in the immunological subtype only. In contrast, LOH at 3p14, 17p13 and mutations in *TP53* were significantly associated with oral cancer development in the classical subtype. Interestingly, the classical subtype of oral premalignant lesions was characterized by an overexpression of EGFR. Even though the earlier described studies on EGFR inhibition were not successful, the classical subtype might still benefit most from this treatment.

In summary, although the above-mentioned treatment options are elegant and very promising, they are not without toxicity and in fact are only applicable if there are better methods available to identify the patients with high-risk precancerous fields. In patients with precancerous fields at high-risk for malignant transformation, some toxicity of the applied drugs will be outweighed.

## 4. Conclusions and Future Directions

The first and most critical step in head and neck cancer surgery is to completely remove the tumor, and residual cancer cells identified by microscopic examination should be treated by postoperative radiotherapy or postoperative chemoradiotherapy, eventually supplemented with immunotherapies or targeted approaches. However, cancer cells in the surgical margins may escape detection under the microscope and when remaining in the patient, cause recurrent disease. These residual cancer cells are missed when examining the resection specimen due to undersampling, the same problem that seems to hamper molecular methods. Sensitive detection of residual cancer cells in the margins, wound bed or elsewhere is key to enable optimal personalized post-operative management, but the problem of undersampling is not easily solved as only a few tumor cells are present in large tissue volumes. Analysis of liquid biopsies might overcome this, although undersampling as a problem may then be substituted for dilution.

A second problematic issue relates to the precancerous fields that precede these tumors and that may stay behind unnoticed. When these undergo progression, a local recurrence or second primary tumor will be the result. Diagnosis of the high-risk fields in the margins is still not standardized, but dysplasia scoring and genetic analysis are able to identify the high-risk changes. The key here is an effective intervention. Radiotherapy is not indicated and as the precancerous changes are mostly not visible, resection is not an option. What is more, autofluorescence-guided resection did not reduce recurrence rates. Hence, drugs need to be selected for systemic interventions, and a few candidates are now on the shelf awaiting clinical trials.

## Figures and Tables

**Table 1:** Overview of a number of detection methods for minimal residual disease in surgical margins or liquid biopsies.

Method	Refs.	Key Findings
*TP53*	[14]	Detection of mutant *TP53* in the surgical margins was associated with a high risk of locoregional recurrence *TP53* is an early genetic change in head and neck carcinogenesis and also detect precancerous fields in the superficial margins Post-operative management of residual cancer and precancerous changes is very different, and the distinction needs to be made.
hLy6D	[16]	hLy6D is a gene that is highly and specifically expressed in squamous cells Undersampling remains the major problem Latest prospective study failed to demonstrate prognostic relevance of hLy6D detection
Methylation markers	[17,19]	Methylation markers analyzed by PCR may be very sensitive, specificity is a major issue Additionally, methylation markers may not be able to overcome the undersampling problem in margin analysis
ctDNA	[22,23,24,25,26,27,28,29,30,31,32,33]	Preferred technique for ctDNA detection is Next Generation Sequencing with high read coverage ctDNA is present in low concentrations, the detection methods must be very sensitive for detection of mutations at mutant allele fractions of <0,1% In other malignancies such as lung, breast, colon and bladder cancer, ctDNA was shown to be clinically relevant To reduce false positive results, clonal hematopoiesis of indeterminate potential (CHIP) filtering should be performed or the index tumors need to be sequenced
Cell-free methylated DNA	[18]	Cell-free methylated DNA immunoprecipitation and high output sequencing (cfMeDIP-seq) applied for genome wide bisulfite-free plasma DNA methylation profiling is promising novel development The technique showed an improved limit of detection compared to ultradeep hybrid capture based sequencing
miRNA expression	[34]	Different combinations of three miRNAs were expressed in the saliva of OSCC patients at high levels compared to controls. This method could effectively stratify patients according to their likelihood of relapse.

**Table 2:** Overview of a number of detection and visualization methods for field cancerization.

Method	Refs.	Key Findings
Dysplasia grading	[43]	Dysplasia is the most consistent predictor of malignant transformation of leukoplakia, particularly when ‘differentiated dysplasia’ is added Dysplasia in the surgical margins is not very predictive for local relapse. Only severe dysplasia is considered for postoperative management. Diagnosis of differentiated dysplasia may change the picture in the future.
LOH analysis of biopsy specimen	[47,48]	Loss of heterozygosity by microsatellite markers at 9p, 3p and 17p was used to describe the process of field cancerization in genetic terms Genetically altered fields were identified in 36% of the patients, and, importantly, in 25% of patients these extended into the surgical margins Approximately half of the local recurrences and half of the second primary tumors in the same or adjacent anatomical area relate to field cancerization Loss of heterozygosity in the margins is a predictor of local relapse
eIF4e	[49]	Eukaryotic translocation initiation factor 4E (eIF4E) can be determined by immunohistochemical analysis, and the expression level of eIF4E was more predictive for local relapse than p53 immunostaining in surgical margins Only a limited of studies reported on this method for detection of field cancerization.
Narrow band Imaging (NBI)	[50,51]	Accurate detection for less severe dysplastic stages was possible, suggesting a potential for surveillance using NBI NBI is fast and optional for intraoperative use Limitations are depth of penetration
Brush biopsy and exfoliative cytology	[54]	Cytology remains an interesting options for the detection premalignant lesions, sampling can take place at the outpatient clinic Sensitivity and specificity meets limitations Has only been tested for leukoplakia lesion and tumor detection
Brush biopsy and genetic analysis	[53]	Adequate detection of a precancerous field using exfoliated cells removed by a brush, using loss of heterozygosity analysis Sensitivity is limited when using loss of heterozygosity analysis Future results with next generation sequencing may be more promising
Brush biopsy Methylation markers	[46]	Quantitative bisulfite NGS analysis could be a highly sensitive and specific method to detect early OSCC starting from non-invasive, easy-to-perform brush sampling.

## References

[1] Bray F., Ferlay J., Soerjomataram I., Siegel R.L., Torre L.A., Jemal A. (2018). Global cancer statistics 2018: GLOBOCAN estimates of incidence and mortality worldwide for 36 cancers in 185 countries. CA Cancer J. Clin..

[2] Siegel R.L., Miller K.D., Jemal A. (2019). Cancer statistics, 2019. CA Cancer J. Clin..

[3] Castellsagué X., Alemany L., Quer M., Halec G., Quirós B., Tous S., Clavero O., Alòs L., Biegner T., Szafarowski T. (2016). hpv involvement in head and neck cancers: Comprehensive assessment of biomarkers in 3680 patients. J. Natl. Cancer Instit..

[4] Leemans C.R., Snijders P.J.F., Brakenhoff R.H. (2018). The molecular landscape of head and neck cancer. Nat. Rev. Cancer.

[5] Bonner J.A., Harari P.M., Giralt J., Azarnia N., Shin D.M., Cohen R.B., Jones C.U., Sur R., Raben D., Jassem J. (2006). Radiotherapy plus cetuximab for squamous-cell carcinoma of the head and neck. N. Engl. J. Med..

[6] Machiels J.P., René Leemans C., Golusinski W., Grau C., Licitra L., Gregoire V. (2020). Squamous cell carcinoma of the oral cavity, larynx, oropharynx and hypopharynx: EHNS-ESMO-ESTRO clinical practice guidelines for diagnosis, treatment and follow-up 2020. Ann. Oncol..

[7] Johnson D.E., Burtness B., Leemans C.R., Lui V.W.Y., Bauman J.E., Grandis J.R. (2020). Head and neck squamous cell carcinoma. Nat. Rev. Dis. Prim..

[8] Van Nieuwenhuizen A.J., Buffart L.M., Langendijk J.A., Vergeer M.R., Voortman J., Leemans C.R., Verdonck-de Leeuw I.M. (2021). Health-related quality of life and overall survival: A prospective study in patients with head and neck cancer treated with radiotherapy. Qual. Life Res. Int. J. Qual. Life Aspects Treatment Care Rehabil..

[9] Rohde M., Rosenberg T., Pareek M., Nankivell P., Sharma N., Mehanna H., Godballe C. (2020). Definition of locally recurrent head and neck squamous cell carcinoma: A systematic review and proposal for the Odense–Birmingham definition. Eur. Arch. Oto Rhino. Laryngol..

[10] Evans M., Beasley M. (2018). Target delineation for postoperative treatment of head and neck cancer. Oral Oncol..

[11] Leemans C.R., Braakhuis B.J., Brakenhoff R.H. (2011). The molecular biology of head and neck cancer. Nat. Rev. Cancer..

[12] Ghosh-Laskar S., Mummudi N., Rangarajan V., Purandare N., Gupta T., Budrukkar A., Murthy V., Agarwal J.P. (2019). Prognostic value of response assessment fluorodeoxyglucose positron emission tomography-computed tomography scan in radically treated squamous cell carcinoma of head and neck: Long-term results of a prospective study. J. Cancer Res. Ther..

[13] Helsen N., Roothans D., Van Den Heuvel B., Van den Wyngaert T., Van den Weyngaert D., Carp L., Stroobants S. (2017). 18F-FDG-PET/CT for the detection of disease in patients with head and neck cancer treated with radiotherapy. PLoS ONE.

[14] Li M.M., Puram S.V., Silverman D.A., Old M.O., Rocco J.W., Kang S.Y. (2019). Margin analysis in head and neck cancer: State of the art and future directions. Ann. Surg. Oncol..

[15] Irani S. (2020). NEW insights into oral cancer-risk factors and prevention: A review of literature. Int. J. Prev. Med..

[16] Mes S.W., Leemans C.R., Brakenhoff R.H. (2016). Applications of molecular diagnostics for personalized treatment of head and neck cancer: State of the art. Expert Rev. Molec. Diagn..

[17] Thomas Robbins K., Triantafyllou A., Suárez C., López F., Hunt J.L., Strojan P., Williams M.D., Braakhuis B.J.M., de Bree R., Hinni M.L. (2019). Surgical margins in head and neck cancer: Intra- and postoperative considerations. Auris Nasus Larynx.

[18] Liu M.C., Oxnard G.R., Klein E.A., Swanton C., Seiden M.V., Liu M.C., Oxnard G.R., Klein E.A., Smith D., Richards D. (2020). Sensitive and specific multi-cancer detection and localization using methylation signatures in cell-free DNA. Ann. Oncol..

[19] Shen S.Y., Singhania R., Fehringer G., Chakravarthy A., Roehrl M.H.A., Chadwick D., Zuzarte P.C., Borgida A., Wang T.T., Li T. (2018). Sensitive tumour detection and classification using plasma cell-free DNA methylomes. Nature.

[20] Wan J.C.M., Massie C., Garcia-Corbacho J., Mouliere F., Brenton J.D., Caldas C., Pacey S., Baird R., Rosenfeld N. (2017). Liquid biopsies come of age: Towards implementation of circulating tumour DNA. Nat. Rev. Cancer.

[21] Mes S.W., Brink A., Sistermans E.A., Straver R., Oudejans C.B.M., Poell J.B., Leemans C.R., Brakenhoff R.H. (2020). Comprehensive multiparameter genetic analysis improves circulating tumor DNA detection in head and neck cancer patients. Oral Oncol..

[22] Chaudhuri A.A., Chabon J.J., Lovejoy A.F., Newman A.M., Stehr H., Azad T.D., Khodadoust M.S., Esfahani M.S., Liu C.L., Zhou L. (2017). Early detection of molecular residual disease in localized lung cancer by circulating tumor dna profiling. Cancer Discov..

[23] Hellmann M.D., Nabet B.Y., Rizvi H., Chaudhuri A.A., Wells D.K., Dunphy M.P.S., Chabon J.J., Liu C.L., Hui A.B., Arbour K.C. (2020). Circulating tumor DNA analysis to assess risk of progression after long-term response to PD-(L)1 blockade in NSCLC. Clin. Cancer Res. Off. J. Am. Assoc. Cancer Res..

[24] Tie J., Cohen J.D., Wang Y., Li L., Christie M., Simons K., Elsaleh H., Kosmider S., Wong R., Yip D. (2019). Serial circulating tumour DNA analysis during multimodality treatment of locally advanced rectal cancer: A prospective biomarker study. Gut.

[25] Tie J., Wang Y., Tomasetti C., Li L., Springer S., Kinde I., Silliman N., Tacey M., Wong H.L., Christie M. (2016). Circulating tumor DNA analysis detects minimal residual disease and predicts recurrence in patients with stage II colon cancer. Sci. Trans. Med..

[26] Abbosh C., Birkbak N.J., Wilson G.A., Jamal-Hanjani M., Constantin T., Salari R., Le Quesne J., Moore D.A., Veeriah S., Rosenthal R. (2017). Phylogenetic ctDNA analysis depicts early-stage lung cancer evolution. Nature.

[27] Garcia-Murillas I., Schiavon G., Weigelt B., Ng C., Hrebien S., Cutts R.J., Cheang M., Osin P., Nerurkar A., Kozarewa I. (2015). Mutation tracking in circulating tumor DNA predicts relapse in early breast cancer. Sci. Trans. Med..

[28] Dudley J.C., Schroers-Martin J., Lazzareschi D.V., Shi W.Y., Chen S.B., Esfahani M.S., Trivedi D., Chabon J.J., Chaudhuri A.A., Stehr H. (2019). Detection and surveillance of bladder cancer using urine tumor DNA. Cancer Discov..

[29] Galot R., van Marcke C., Helaers R., Mendola A., Goebbels R.M., Caignet X., Ambroise J., Wittouck K., Vikkula M., Limaye N. (2020). Liquid biopsy for mutational profiling of locoregional recurrent and/or metastatic head and neck squamous cell carcinoma. Oral Oncol..

[30] Schirmer M.A., Beck J., Leu M., Oellerich M., Rave-Fränk M., Walson P.D., Schütz E., Canis M. (2018). Cell-free plasma DNA for disease stratification and prognosis in head and neck cancer. Clin. Chem..

[31] Schwaederle M., Chattopadhyay R., Kato S., Fanta P.T., Banks K.C., Choi I.S., Piccioni D.E., Ikeda S., Talasaz A., Lanman R.B. (2017). Genomic alterations in circulating tumor DNA from diverse cancer patients identified by next-generation sequencing. Cancer Res..

[32] Wang Y., Springer S., Mulvey C.L., Silliman N., Schaefer J., Sausen M., James N., Rettig E.M., Guo T., Pickering C.R. (2015). Detection of somatic mutations and HPV in the saliva and plasma of patients with head and neck squamous cell carcinomas. Sci. Trans. Med..

[33] Razavi P., Li B.T., Brown D.N., Jung B., Hubbell E., Shen R., Abida W., Juluru K., De Bruijn I., Hou C. (2019). High-intensity sequencing reveals the sources of plasma circulating cell-free DNA variants. Nat. Med..

[34] Romani C., Salviato E., Paderno A., Zanotti L., Ravaggi A., Deganello A., Berretti G., Gualtieri T., Marchini S., D’Incalci M. (2021). Genome-wide study of salivary miRNAs identifies miR-423-5p as promising diagnostic and prognostic biomarker in oral squamous cell carcinoma. Theranostics.

[35] Ferris R.L., Geiger J.L., Trivedi S., Schmitt N.C., Heron D.E., Johnson J.T., Kim S., Duvvuri U., Clump D.A., Bauman J.E. (2016). Phase II trial of post-operative radiotherapy with concurrent cisplatin plus panitumumab in patients with high-risk, resected head and neck cancer. Ann. Oncol..

[36] Harrington K.J., Ferris R.L., Blumenschein G., Colevas A.D., Fayette J., Licitra L., Kasper S., Even C., Vokes E.E., Worden F. (2017). Nivolumab versus standard, single-agent therapy of investigator’s choice in recurrent or metastatic squamous cell carcinoma of the head and neck (CheckMate 141): Health-related quality-of-life results from a randomised, phase 3 trial. Lancet Oncol..

[37] Ferris R.L., Blumenschein G., Fayette J., Guigay J., Colevas A.D., Licitra L., Harrington K., Kasper S., Vokes E.E., Even C. (2016). Nivolumab for recurrent squamous-cell carcinoma of the head and neck. N. Engl. J. Med..

[38] De Boer D.V., Brink A., Buijze M., Stigter-van Walsum M., Hunter K.D., Ylstra B., Bloemena E., Leemans C.R., Brakenhoff R.H. (2019). Establishment and genetic landscape of precancer cell model systems from the head and neck mucosal lining. Molec. Cancer Res. MCR.

[39] Evren I., Brouns E.R., Wils L.J., Poell J.B., Peeters C.F.W., Brakenhoff R.H., Bloemena E., de Visscher J. (2020). Annual malignant transformation rate of oral leukoplakia remains consistent: A long-term follow-up study. Oral Oncol..

[40] Wils L.J., Poell J.B., Evren I., Koopman M.S., Brouns E., de Visscher J., Brakenhoff R.H., Bloemena E. (2020). Incorporation of differentiated dysplasia improves prediction of oral leukoplakia at increased risk of malignant progression. Modern Pathol..

[41] Braakhuis B.J., Tabor M.P., Kummer J.A., Leemans C.R., Brakenhoff R.H. (2003). A genetic explanation of Slaughter’s concept of field cancerization: Evidence and clinical implications. Cancer Res..

[42] Van Zeeburg H.J., Graveland A.P., Brink A., Nguyen M., Leemans C.R., Bloemena E., Braakhuis B.J., Brakenhoff R.H. (2013). Generation of precursor cell lines from preneoplastic fields surrounding head and neck cancers. Head Neck..

[43] Simple M., Suresh A., Das D., Kuriakose M.A. (2015). Cancer stem cells and field cancerization of oral squamous cell carcinoma. Oral Oncol..

[44] Roy R., Singh R., Chattopadhyay E., Ray A., Sarkar N., Aich R., Paul R.R., Pal M., Roy B. (2016). MicroRNA and target gene expression based clustering of oral cancer, precancer and normal tissues. Gene.

[45] Zhao C., Zou H., Zhang J., Wang J., Liu H. (2018). An integrated methylation and gene expression microarray analysis reveals significant prognostic biomarkers in oral squamous cell carcinoma. Oncol. Rep..

[46] Morandi L., Gissi D., Tarsitano A., Asioli S., Gabusi A., Marchetti C., Montebugnoli L., Foschini M.P. (2017). CpG location and methylation level are crucial factors for the early detection of oral squamous cell carcinoma in brushing samples using bisulfite sequencing of a 13-gene panel. Clin. Epigenetics.

[47] Califano J., Westra W.H., Meininger G., Corio R., Koch W.M., Sidransky D. (2000). Genetic progression and clonal relationship of recurrent premalignant head and neck lesions. Clin. Cancer Res. Off. J. Am. Assoc. Cancer Res..

[48] Tabor M.P., Brakenhoff R.H., van Houten V.M., Kummer J.A., Snel M.H., Snijders P.J., Snow G.B., Leemans C.R., Braakhuis B.J. (2001). Persistence of genetically altered fields in head and neck cancer patients: Biological and clinical implications. Clin. Cancer Res. Off. J. Am. Assoc. Cancer Res..

[49] Singh J., Jayaraj R., Baxi S., Mileva M., Skinner J., Dhand N.K., Thomas M. (2016). Immunohistochemical expression levels of p53 and eIF4E markers in histologically negative surgical margins, and their association with the clinical outcome of patients with head and neck squamous cell carcinoma. Molec. Clin. Oncol..

[50] Vu A.N., Matias M., Farah C.S. (2015). Diagnostic accuracy of Narrow Band Imaging for the detection of oral potentially malignant disorders. Oral Dis..

[51] Ottaviani G., Gobbo M., Rupel K., D’Ambros M., Perinetti G., Di Lenarda R., Martinelli V., Bussani R., Tirelli G., Lodi G. (2016). The diagnostic performance parameters of Narrow Band Imaging: A preclinical and clinical study. Oral Oncol..

[52] Poh C.F., Anderson D.W., Durham J.S., Chen J., Berean K.W., MacAulay C.E., Rosin M.P. (2016). Fluorescence Visualization-Guided Surgery for Early-Stage Oral Cancer. JAMA Otolaryngol. Head Neck Surg..

[53] Saidak Z., Lailler C., Testelin S., Chauffert B., Clatot F., Galmiche A. (2021). Contribution of Genomics to the Surgical Management and Study of Oral Cancer. Ann. Surg. Oncol..

[54] Gupta S., Shah J.S., Parikh S., Limbdiwala P., Goel S. (2014). Clinical correlative study on early detection of oral cancer and precancerous lesions by modified oral brush biopsy and cytology followed by histopathology. J. Cancer Res. Ther..

[55] Mogedas-Vegara A., Hueto-Madrid J.A., Chimenos-Küstner E., Bescós-Atín C. (2016). Oral leukoplakia treatment with the carbon dioxide laser: A systematic review of the literature. J. Cranio-Maxillo-Facial Surg. Off. Publ. Eur. Assoc. Cranio-Maxillo-Facial Surg..

[56] Durham J.S., Brasher P., Anderson D.W., Yoo J., Hart R., Dort J.C., Seikaly H., Kerr P., Rosin M.P., Poh C.F. (2020). Effect of fluorescence visualization-guided surgery on local recurrence of oral squamous cell carcinoma: A randomized clinical trial. JAMA Otolaryngol. Head Neck Surg..

[57] Albini A., DeCensi A., Cavalli F., Costa A. (2016). Cancer Prevention and Interception: A New Era for Chemopreventive Approaches. Clin. Cancer Res. Off. J. Am. Assoc. Cancer Res..

[58] Bhatia A., Burtness B. (2017). Novel molecular targets for chemoprevention in malignancies of the head and neck. Cancers.

[59] Grigolato R., Bizzoca M.E., Calabrese L., Leuci S., Mignogna M.D., Lo Muzio L. (2020). Leukoplakia and Immunology: New chemoprevention landscapes?. Int. J. Molec. Sci..

[60] Van Zandwijk N., Dalesio O., Pastorino U., de Vries N., van Tinteren H. (2000). Euroscan, a randomized trial of vitamin A and N-acetylcysteine in patients with head and neck cancer or lung cancer. For the European organization for research and treatment of cancer head and neck and lung cancer cooperative groups. J. Natl. Cancer Instit..

[61] Khan Z., Epstein J.B., Marur S., Gillespie M.B., Feldman L., Tsai H.L., Zhang Z., Wang H., Sciubba J., Ferris R. (2016). Cetuximab activity in dysplastic lesions of the upper aerodigestive tract. Oral Oncol..

[62] William W.N., Papadimitrakopoulou V., Lee J.J., Mao L., Cohen E.E., Lin H.Y., Gillenwater A.M., Martin J.W., Lingen M.W., Boyle J.O. (2016). Erlotinib and the risk of oral cancer: The erlotinib prevention of oral cancer (EPOC) randomized clinical trial. JAMA Oncol..

[63] De Boer D.V., Martens-de Kemp S.R., Buijze M., Stigter-van Walsum M., Bloemena E., Dietrich R., Leemans C.R., van Beusechem V.W., Braakhuis B.J.M., Brakenhoff R.H. (2017). Targeting PLK1 as a novel chemopreventive approach to eradicate preneoplastic mucosal changes in the head and neck. Oncotarget.

[64] Goan Y.G., Liu P.F., Chang H.W., Chen H.C., Chen W.C., Kuo S.M., Lee C.H., Shu C.W. (2019). Kinome-wide screening with small interfering RNA identified polo-like Kinase 1 as a key regulator of proliferation in oral cancer cells. Cancers.

[65] Schmitz S., Machiels J.P. (2016). Targeting the tumor environment in squamous cell carcinoma of the head and neck. Curr. Treat. Options Oncol..

[66] Wang J., Xie T., Wang B., William W.N., Heymach J.V., El-Naggar A.K., Myers J.N., Caulin C. (2017). PD-1 blockade prevents the development and progression of carcinogen-induced oral premalignant lesions. Cancer Prev. Res..

[67] Monteiro de Oliveira Novaes J.A., Hirz T., Guijarro I., Nilsson M., Pisegna M.A., Poteete A., Barsoumian H.B., Fradette J.J., Chen L.N., Gibbons D.L. (2021). Targeting of CD40 and PD-L1 pathways inhibits progression of oral premalignant lesions in a carcinogen-induced model of oral squamous cell carcinoma. Cancer Prev. Res..

[68] Foy J.P., Bertolus C., Ortiz-Cuaran S., Albaret M.A., Williams W.N., Lang W., Destandau S., Souza G., Sohier E., Kielbassa J. (2018). Immunological and classical subtypes of oral premalignant lesions. Oncoimmunology.

[69] Carenzo A., Serafini M.S., Roca E., Paderno A., Mattavelli D., Romani C., Saintigny P., Koljenović S., Licitra L., De Cecco L. (2020). Gene expression clustering and selected head and neck cancer gene signatures highlight risk probability differences in oral premalignant lesions. Cells.

